# Characteristics and risk factors of preterm births in a tertiary center in Lagos, Nigeria

**DOI:** 10.11604/pamj.2016.24.1.8382

**Published:** 2016-05-01

**Authors:** Azeez Butali, Chinyere Ezeaka, Osayame Ekhaguere, Nancy Weathers, Jenna Ladd, Iretiola Fajolu, Christopher Esezobor, Christian Makwe, Bukola Odusanya, Rose Anorlu, Wasiu Adeyemo, Edna Iroha, Mathias Egri-Okwaji, Prisca Adejumo, Lawal Oyeneyin, Moses Abiodun, Bolaji Badejoko, Kelli Ryckman

**Affiliations:** 1 Department of Oral Pathology, Radiology and Medicine, College of Dentistry, University of Iowa, IA, USA; 2 Department of Pediatrics, College of Medicine, University of Lagos, Nigeria; 3 Department of Pediatrics, University of Iowa, IA, USA; 4 Department of Epidemiology, College of Public Health, University of Iowa, IA, USA; 5 Department of Obstetrics and Gynecology, College of Medicine, University of Lagos, Nigeria; 6 Department of Oral and Maxillofacial Surgery, College of Medicine, University of Lagos, Nigeria; 7 Department of Nursing, University of Ibadan, Nigeria; 8 Department of Obstetrics and Gynecology, Mother and Child Hospital Ondo, Ondo, Nigeria; 9 Department of Pediatrics, Mother and Child Hospital Ondo, Ondo, Nigeria

**Keywords:** Preterm birth, Nigeria, not registered for antenatal care

## Abstract

**Introduction:**

Preterm birth is a dire complication of pregnancy that poses huge long-term medical and financial burdens for affected children, their families, and the health care system. The aim of the present study was to identify characteristics associated with preterm births at the Lagos University Teaching Hospital (LUTH), Lagos, Nigeria from 2011 to 2013.

**Methods:**

We obtained Information from 5,561 maternal, fetal/neonatal and obstetric records from the labor ward. We excluded delivery at less than 22 weeks (0.25%), post-term birth at ≥42 weeks gestation (1.3%), and unknown gestation (1.4%). Additionally, we excluded records of multiple births (5.4%) and stillbirths (8.3%) leaving 4,691 records of singleton live-births for analysis. Logistic regression analysis was performed comparing preterm birth (22-36 weeks gestation) to term birth (37-41 weeks gestation). Multiple variable models adjusting for maternal age, parity, fetal position, delivery method and booking status were also evaluated. Multinomial regression was used to identify characteristics associated with preterm birth (PTB) defined as early PTB (22-31 weeks gestation), moderate PTB (32-34 weeks gestation), late PTB (35-36 weeks gestation), compared to term birth (37-41 completed weeks gestation).

**Results:**

From our data, 16.8% of the singleton live-birth deliveries were preterm (<37 weeks gestation). Of these, 4.7% were early (22-31 weeks), 4.5% were moderate (32-34 weeks) and 7.7% were late (35-36) PTBs. Older maternal age (≥35 years) [odds ratio (OR) = 1.41], hypertension (OR = 3.44) and rupture of membranes (OR = 4.03) were significantly associated with increased odds of PTB. Women being treated for the prevention of mother-to-child transmission of HIV were at a significantly decreased risk for PTB (OR = 0.70). Sixteen percent of women in this cohort were not registered for antenatal care in LUTH. These non-registered subjects had significantly greater odds of all categories of PTB, including early (odds ratio (OR) = 20.8), moderate (OR = 8.68), and late (OR = 2.15).

**Conclusion:**

PTB and risks for PTB remain high in Nigeria. We recommend that any high risk pregnancy should be referred to a tertiary center for prenatal care in order to significantly reduce adverse birth outcomes such as PTBs.

## Introduction

Preterm birth (PTB), defined as birth of an infant before 37 post-menstrual weeks (and after 20 weeks gestation), is a dire complication of pregnancy that incurs long-term medical and financial burdens for affected children, their families, and the health care system. The annual societal burden of PTB is estimated to be about $26.2 billion in the United States [1]. Obstetric history of past PTB and cervical length measurements are two of the most accurate and most widely used predictors of PTB; however, these predictors are inconsistently applied in developed countries and often impracticable in low-income settings. In the past 20 years, there has been a global increase in the frequency of PTB [2, 3]. In Africa the frequency of pregnancies affected by PTB is increasing with a prevalence of 7.4%, with 68.2% of PTBs occurring spontaneously [4]. In spite of improvements in neonatal care, PTB is now the biggest single cause of death and long-term disability worldwide [5]. PTB results in over 1 million deaths per year globally, with over 90% of these in low- and middle-income countries [6, 7]. Globally, more than 75 million person years of productive life are lost because of PTB, which is mainly a consequence of early mortality and lifelong disability [8]. Currently, prematurity has been noted to be the commonest cause of under-five mortality globally, well above pneumonias and malaria [5]. Furthermore, the earlier in gestation that PTB occurs, the greater the risks of adverse outcomes; however, infants born late preterm (35-36 weeks gestation) still have considerably higher morbidity and mortality compared to their term counterparts. The main triggers for PTB include a wide range of interacting genetic and environmental factors. Nigeria is the most populous country in Africa, with a population of over 170 million people as of 2014 [9]. In 2014, the total fertility rate and crude birth rate were reported to be 5.5 and 39 births/1000, respectively [10]. Child bearing takes place mainly at an early age and reports show that 23% of women aged 15-19 have already begun childbearing and about one-third (32%) of women aged 20-49 have had a birth by age 18 [10]. Infant mortality in Nigeria is very high, ranked 74^th^ in the world in 2013 [11]. This is primarily due to PTBs, pregnancy complications such as preeclampsia and eclampsia, infections such as Human immuno-deficiency virus (HIV), and malnutrition. Furthermore, Nigeria currently has the highest number of newborn deaths in Africa, and the second highest in the world (coming only after India) [12, 13]. About 270,000 children in Nigeria per year die within the first month of life as a direct consequence of PTB and low birth weight, perinatal asphyxia, and, infections [12, 13]. Considering that over 50% of births are delivered outside the hospital [14, 15], it is possible that these women received little or no perinatal care, a key element in maternal and child health. Hospitals in Nigeria are classified into the three-tier health system consisting of primary, secondary, and tertiary level care, each having varying degrees of capacity and oversight roles. The tertiary hospitals are major referral center with many of the pregnant mothers admitted in emergency wards as “non-booked”, most referred late to the hospital due to pregnancy complications. In the present study, we describe in detail maternal, fetal/neonatal and obstetric characteristics associated with PTB at the Lagos University Teaching Hospital, a tertiary hospital in Lagos, Nigeria.

## Methods

Data for this study was obtained from the labor ward records of the Lagos University Teaching Hospital (LUTH), Surulere, Lagos, Nigeria. The following categories were obtained and entered: age of mother, booking status, parity, previous miscarriages, multiple or singleton gestation, spontaneous or induced labor, gestational age (this was calculated using a combination of the LMP and best obstetrics i.e. an ultrasound is requested at the time of registration for all booked patients), blood group, Rhesus positive or negative, presentation and position of the fetus, type of delivery (vaginal, Caesarean, or assisted (i.e. forceps and vacuum delivery), delivery date, volume of blood loss, placenta (complete/ incomplete), placenta weight, sex of infant, weight of infant, whether the neonate was alive or dead at birth, and American Paediatric Gross Assessment Record (APGAR) score. Additional information was abstracted from the clinic notes on pregnancy complications including data on the presence of the following factors: hypertension (defined as gestational or chronic including preeclampsia and eclampsia), diabetes (defined as gestational or chronic), sickle cell anemia, prevention of maternal to child transmission of HIV (PMTCT), placental previa, placental abruption, preterm and prolonged rupture of membranes, prolonged labor, and cephalopelvic disproportion. There were 5561 registered births from January 2011 through December 2013 for data analysis. Our primary focus was characteristics of PTBs; therefore, we excluded delivery at less than 22 weeks (0.25%), post-term birth at ≥42 weeks gestation (1.3%), and unknown gestation (1.4%). Additionally, we excluded records of multiple births (5.4%) and stillbirths (8.3%) focusing only on live-born singleton deliveries leaving 4,691 records for analysis. Logistic regression analysis was performed to compare PTB (22-36 weeks gestation) to term birth (37-41 completed weeks gestation). Multinomial regression was used to identify characteristics associated with PTB defined by four categories: early PTB (22-31 weeks gestation), moderate PTB (32-34 weeks gestation), late PTB (35-36 weeks gestation), compared to term birth (37-41 weeks gestation). Maternal conditions with small numbers were not included in the PTB sub-category analysis but in the broad PTB versus Term comparison. Statistical analyses were performed using Stata/SE 12.1 (Stata Corp, College Station, TX, USA).

## Results

PTB was prevalent in this population, with 790(16.8%) of the deliveries occurring preterm (<37 weeks gestation). Of these, 218 (4.7%) were early (22-31 weeks) PTBs, 212 (4.5%) were moderate PTBs (32-34 weeks) and 360 (7.7%) were late (35-36 weeks) PTBs. Overall, in multiple variable models, PTB was significantly associated with older (>35) maternal age (15-24), being non-booked (non-registered) for antenatal care at the study site (LUTH), surgical or assisted delivery, any hypertension in addition to preeclampsia and eclampsia, and rupture of membranes (Table 1). Women being treated for the prevention of mother-to-child transmission of HIV were at a significantly decreased risk for PTB. Sixteen percent of women in this cohort were non-booked for antenatal care and delivery at the hospital but were referred and brought in during the course of their labour and delivery, due to onset of complications (Table 1). In relation to infant characteristics, as expected, infants born preterm were more likely to have low birth weight and lower APGAR scores at 1 and 5 minutes than their term counterparts (Table 2). When examining characteristics and risk factors across categories of PTB and including adjustment for relevant covariates, older maternal age (≥35) was associated with increased odds of moderate and late but not early PTB (Table 3). It is noteworthy that mothers not booked at LUTH (non-registered) were at a significantly greater odds of all categories of PTB including early (OR = 20.8), moderate (OR = 8.68) and late (OR = 2.15) (Table 3). Women who had any degree of PTB were more likely to deliver by either assisted delivery or Caesarean section (Table 1 and Table 3); however, caesarean section was not associated with early PTB and assisted delivery was not associated with late PTB. Women being treated for the prevention of mother to child transmission of HIV were at a significantly decreased risk for moderate (OR = 0.37) PTB but not early or late PTB (Table 3).

**Table 1:** Characteristics and risk factors of PTB in singleton live-births, N = 4,691

Characteristic	PTB (<37 weeks) N = 790	Term (37-41 weeks) N = 3,901	Unadjusted OR (95% CI)	Adjusted OR* (95% CI)
**Maternal Age**				
15–24	64 (8.1)	253 (6.5)	1.45 (1.08-1.94)	1.14 (0.82-1.60)
25–34	488 (61.8)	2794 (71.6)	1.00 (Referent)	1.00 (Referent)
≥35	238 (30.1)	848 (21.7)	1.61 (1.35-1.91)	1.41 (1.15-1.74)
Missing	0 (0)	6 (0.2)	N/A	N/A
Parity				
0	291 (36.8)	1373 (35.2)	1.00 (Referent)	1.00 (Referent)
1	163 (20.6)	1144 (29.3)	0.67 (0.55-0.83)	0.73 (0.58-0.92)
2	179 (22.7)	755 (19.4)	1.12 (0.91-1.37)	1.23 (0.97-1.56)
3	88 (11.1)	339 (8.7)	1.22 (0.94-1.60)	1.26 (0.93-1.71)
4 +	51 (6.5)	203 (5.2)	1.19 (0.85-1.65)	1.11 (0.75-1.62)
Missing	18 (2.3)	87 (2.2)	N/A	N/A
**Previous Miscarriages**				
0	440 (55.7)	2179 (55.9)	1.00 (Referent)	1.00 (Referent)
1	139 (17.6)	787 (20.2)	0.87 (0.71-1.08)	0.97 (0.77-1.22)
2	101 (12.8)	451 (11.6)	1.11 (0.87-1.41)	1.19 (0.91-1.55)
3 +	88 (11.1)	369 (9.5)	1.18 (0.92-1.52)	1.14 (0.85-1.52)
Missing	22 (2.8)	115 (3.0)		N/A
**Booked in Hospital**				
Yes	410 (51.9)	3350 (85.9)	1.00 (Referent)	1.00 (Referent)
No	328 (41.5)	407 (10.4)	6.58 (5.51-7.87)	6.13 (5.08-7.40)
Missing	52 (6.6)	144 (3.7)	N/A	N/A
**Fetal Position**				
Cephalic	725 (91.8)	3711 (95.1)	1.00 (Referent)	1.00 (Referent)
Breech	62 (7.9)	177 (4.5)	1.79 (1.33-2.42)	1.14 (0.80-1.63)
Missing	3 (0.4)	13 (0.3)	N/A	N/A
**Delivery Mode**				
Vaginal Delivery	256 (32.4)	2009 (51.5)	1.00 (Referent)	1.00 (Referent)
Caesarean Section	509 (64.4)	1850 (47.4)	2.16 (1.83-2.54)	1.61 (1.34-1.94)
Assisted	22 (2.8)	38 (1.0)	4.54 (2.65-7.80)	3.62 (1.91-6.86)
Missing	3 (0.4)	4 (0.1)	N/A	N/A
**Maternal Conditions**				
Sickle Cell Anaemia	12 (1.5)	45 (1.1)	1.32 (0.70-2.51)	1.42 (0.71-2.85)
PMTCT	58 (7.3)	397 (10.2)	0.70 (0.52-0.93)	0.70 (0.51-0.97)
Diabetes	5 (0.6)	47 (1.2)	0.52 (0.21-1.32)	0.47 (0.16-1.35)
Any Hypertension	175 (22.2)	162 (4.2)	6.57 (5.22-8.27)	3.44 (2.60-4.55)
Preeclampsia	89 (11.3)	63 (1.6)	7.73 (5.55-10.79)	3.49 (2.36-5.17)
Eclampsia	44 (5.6)	24 (0.6)	9.53 (5.76-15.76)	3.41 (1.92-6.05)
Uterine Rupture	1 (0.1)	6 (0.2)	0.82 (0.10-6.84)	0.45 (0.04-4.56)
Antepartum Haemorrhage	8 (1.0)	11 (0.3)	3.62 (1.45-9.02)	2.13 (0.73-6.18)
Rupture of Membranes	54 (6.8)	47 (1.2)	6.02 (4.04-8.97)	4.03 (2.54-6.40)

PTB: pre-term births; OR; Odd ration

**Table 2:** Infant characteristics of singleton live-birth preterm births, N = 4,691

Characteristic	PTB (<37 weeks) N = 790	Term (37-41 weeks) N = 3,901	p-value
**Infant Gender**			0.72
Female	377 (47.7)	1892 (48.5)	
Male	411 (52.0)	2006 (51.4)	
Missing	2 (0.3)	3 (0.1)	
**Infant Birth Weight**			<0.001
<1500g	165 (20.9)	7 (0.2)	
1500-2499g	330 (41.8)	185 (4.7)	
2500-3999g	280 (35.4)	3383 (86.7)	
> = 4000g	10 (1.3)	318 (8.2)	
**Infant APGAR at 1 minute**			<0.001
≤3	59 (7.5)	112 (2.9)	
4-7	311 (39.4)	703 (18.0)	
≥8	359 (45.4)	2929 (75.1)	
Missing	61 (7.7)	157 (4.0)	
**Infant APGAR at 5 minutes**			<0.001
≤3	5 (0.6)	9 (0.2)	
4-7	98 (12.4)	169 (4.3)	
≥8	626 (79.2)	3564 (91.4)	
Missing	61 (7.7)	159 (4.1)	

PTB: Pre-term birth

**Table 3:** Comparison of PTB categories in 22 to 41 weeks gestation, N = 4,691

Characteristic	Early PTB vs Term	Moderate PTB vs Term	Late PTB vs Term
**Maternal Age**			
15–24	1.07 (0.63–1.83)	0.99 (0.56–1.75)	1.32 (0.82–2.11)
25–34	1.00 (Referent)	1.00 (Referent)	1.00 (Referent)
≥35	1.17 (0.79–1.74)	1.45 (1.01–2.08)	1.47 (1.13–1.93)
**Parity**			
0	1.00 (Referent)	1.00 (Referent)	1.00 (Referent)
1	0.61 (0.40–0.93)	0.62 (0.41–0.93)	0.89 (0.65–1.22)
2	1.01 (0.65–1.56)	0.92 (0.60–1.41)	1.56 (1.14–2.14)
3	0.98 (0.56–1.73)	0.93 (0.54–1.60)	1.65 (1.12–2.44)
4 +	1.33 (0.69–2.54)	1.24 (0.68–2.26)	0.95 (0.44–1.67)
**Previous Miscarriages**			
0	1.00 (Referent)	1.00 (Referent)	1.00 (Referent)
1	1.05 (0.69–1.59)	0.82 (0.54–1.26)	1.00 (0.74–1.34)
2	1.39 (0.87–2.22)	1.24 (0.79–1.96)	1.07 (0.75–1.52)
3 +	1.38 (0.81–2.33)	1.41 (0.88–2.25)	0.91 (0.61–1.36)
**Booked in Hospital**			
Yes	1.00 (Referent)	1.00 (Referent)	1.00 (Referent)
No	20.84 (14.84–29.25)	8.68 (6.36–11.83)	2.15 (1.60–2.87)
**Fetal Position**			
Cephalic	1.00 (Referent)	1.00 (Referent)	1.00 (Referent)
Breech	1.64 (0.92–2.93)	1.10 (0.61–1.99)	0.94 (0.54–1.67)
**Delivery Mode**			
Vaginal Delivery	1.00 (Referent)	1.00 (Referent)	1.00 (Referent)
Caesarean Section	0.86 (0.61–1.20)	2.06 (1.46–2.91)	1.92 (1.50–2.46)
Assisted	5.15 (2.06–12.89)	4.42 (1.52–12.86)	2.05 (0.70–6.03)
**Maternal Conditions**			
Hypertension	4.15 (2.63–6.55)	4.45 (2.97–6.67)	2.76 (1.90–4.00)
PMTCT	0.44 (0.19–1.04)	0.37 (0.18–0.78)	0.92 (0.63–1.33)

PTB: Pre-term birth

## Discussion

This study adds to the body of evidence provided by other hospital-based studies demonstrating that the rate of PTB is significantly high in Nigeria. It reveals a prevalence rate of 16.8%, which falls outside the 9.5-15.8% prevalence reported by World Health Organization (WHO) for Sub-Saharan Africa [16] but is consistent with previous estimates between 15% and 23% from Nigeria [17–20]. Our single-center study design is similar to the aforementioned studies from Nigeria. However, our center is a larger referral center with a bias for receiving complicated pregnancies that may lead to preterm deliveries. It is well known that the degree of prematurity inversely relates to the likelihood of increased mortality and morbidity. Therefore, our characterization of factors contributing to preterm delivery is stratified according to degree of prematurity to better improve our current understanding of the aetiology and risks for PTB. In addition, the technological, personnel, and financial resources needed to care for a 28 vs a 36 week premature infant differ considerably. Thus, in low-resource settings where the majority of healthcare institutions lack the basic necessities to care for premature neonates and have virtually no infrastructure for prompt referral to tertiary centers, the implication of an early PTB vs a late PTB becomes critical. Because a larger percentage of preterm births are typically moderate to late preterm births, it becomes paramount to describe preterm delivery in terms of strata in order to direct cost-effective resources appropriately [21]. Twenty-three percent of the women in this cohort were older maternal age (≥35 years) with 3.5% of these women being advanced maternal age (≥40 years). Only a small percentage (0.6%) of women were <20 years of age, which is lower than previous reports from sub-Saharan Africa [22]. It has been widely reported that advanced maternal age is associated with PTB [23, 24]. This study reports similar findings as maternal age >35 years was associated with moderate and late PTB. It is therefore possible to consider advanced maternal age as a direct link or a risk marker through its association with age-dependent confounders for preterm delivery. Sixteen percent of women in this cohort were unregistered in our study site and were termed “non-booked mothers”. This is not surprising considering that LUTH is a tertiary hospital and referral center. Being non-booked is significantly associated with all preterm delivery, regardless of severity. Similarly Tucker et al. (2009) [25] reported that non-booked mothers were five times more likely to have preterm delivery (OR = 6.44, 95%, CI: 2.24-18.50) in a study carried out in the United Kingdom (UK). Lack of prenatal care has been demonstrated by studies to negatively affect pregnancy outcome, such as increased maternal mortality, complicated maternal emergency, and perinatal mortality [26, 27]. Our associations of booking status were robust to maternal age, parity, fetal position and delivery method. Hypertension during pregnancy negatively impacts placental blood flow and leads to poor fetal growth and obstetric emergencies, which increase the odds of having a preterm surgical delivery or induced preterm delivery as a lifesaving measure for the mother and fetus.

In several studies maternal hypertension has been shown to be significantly associated with PTB outcomes [28–30]. In the current study, we observed a similar trend in which maternal hypertension was significantly associated with all categories of preterm delivery. A study looking at the biological determinant of late PTB found that placental ischemia as a result of hypertensive diseases in pregnancy increased the odds of late preterm delivery [31]. Other maternal medical conditions associated with increased risk of preterm delivery included placenta previa, placenta abruption, and preterm premature rupture of membranes, all of which are in concordance with other reported studies in the region and globally [16, 17, 19, 29, 30, 32, 33]. Approximately one in ten women in this cohort was infected with HIV and was in treatment to prevent maternal to child transmission (PMTCT). Receiving care for PMTCT significantly reduced the risk of early and moderate preterm delivery but not late preterm delivery. This was the only variable that conferred a reduced risk for preterm delivery. A similar finding was reported by Joseph et al. (2011) [34] in Benin City, Nigeria when they observed that women with HIV/AIDS on highly active antiretroviral therapy had better pregnancy outcomes than non-booked women with HIV. We postulate that these mothers, due to their HIV status and need for prevention of child transmission, may have been booked and thus more committed to their prenatal care and as such received adequate management during pregnancy. This is further supported by a study from Cameroon which found a significantly higher proportion of HIV-positive mothers had on average four antenatal care visits more than HIV-negative mothers [35]. This study also found no increased risk of premature birth in HIV-positive mothers when compared to HIV-negative mothers. This is similar to the findings of a previous study from this center [36]. However, when examining early preterm delivery (22-31 weeks), we still find a moderate protective effect between PMTCT and PTB even after adjustment for booking status (OR = 0.44 95% CI =0.19-1.04). This is contradictory to other studies that found an increased risk of PTB in HIV-positive mothers [19, 37]. The study by Olusanya et al. (2010) [19], however, did not report if these HIV-positive women were on the PMTCT protocol and they also grouped HIV infection with other infections. A meta-analysis which found an increased risk of prematurity and HIV looked at studies from 1983 through1996 [38], a period when much was yet to be understood about HIV transmission as compared to present day, and most of the mothers were not on highly active antiretroviral therapy. We were limited in the current study to assessing only clinical variables that were recorded in the labor and delivery registry which does not contain all previously published clinical variables that can contribute to PTB. Clinical variables such as malaria infection, maternal nutrition status, history of PTBs, and urine group B streptococus status have been shown to contribute considerably to the etiology of PTB [39]. Also, as with many single-centered retrospective studies, it is difficult to generalize these findings to the Nigerian population. Our prevalence rate could be overstated due to selection bias since LUTH is one of the largest referral centers in Lagos State. On the other hand, we may have under-estimated the prevalence rate considering that we excluded multiple gestations which were viable at birth. Furthermore, during the study period there was a span of approximately eight months when the hospital was closed on account of industrial action by medical and non-medical personnel, thus our study population is less than expected. Nonetheless, we provide striking evidence that exists at both ends of the spectrum as it relates to provision and utilization of perinatal care. Perinatal care has been described extensively in the literature as one of the key determinants of maternal and infant health indices. The booking process at the LUTH requires that the pregnant mother formally registers for prenatal care in the first trimester and sometimes second trimester for some mothers. At this registration, routine tests such as blood pressure, urine, blood and scans are carried out. The patient is then assigned to a consultant and is subsequently given routine clinic appointments. These appointments are initially first monthly, then two weekly, and then weekly till delivery. Delivery is planned based on the individual's peculiarity. Unbooked (non-registered) means they did not attend nor register for prenatal services in LUTH but were taken there from another facility as an emergency during labour with antecedent complications. Being non-booked and receiving no prenatal care was the only modifiable maternal characteristic that increased the risk of PTB in all strata. Most non-booked subjects received prenatal care in private, by traditional birth attendants (TBAs), or in primary and secondary centers but were referred late to the tertiary center in emergency with complications. We assumed that these mothers may have been in a high risk category but were not identified, managed, or referred early. Expectant mothers with HIV and PMTCT are categorized as high risk and this knowledge is embodied by both the expectant mothers who are striving to deliver a healthy HIV-free baby and the healthcare team who are striving to prevent the transmission from mother to child. The content of prenatal care geared at identifying at-risk mothers, not the number of visits or how early the first visit, has been proposed to be the key in reducing PTBs [40]. European prenatal care guidelines emphasize prevention of risk and have shown reduced rates of PTBs over time in France [41]. A health care system that is proactive and treats every pregnancy with such delicacy with the understanding that pregnancy and childbirth poses a grave risk to the mother and infant may potentially be able to curb not only the prevalence of PTB and its complications, but also reduce the rate of maternal mortality due to obstetric complications. One possible future study that will be exciting to undertake will be to assess the degree to which the factors examined in the present analysis contribute to PTB considering that PTB itself is a complex interaction between genetic, metabolomics, microbiome, and social factors with difficulty in ascribing causation.

## Conclusion

In conclusion, our data suggest that PTB remains high in Nigeria and is still within ranges that have been reported previously. We also observed that there are modifiable and non-modifiable factors that contribute to PTBs in the study population. Based on our data and evidence from other related studies in this population, we recommend that every pregnancy that is regarded as high risk by the health care system in Nigeria be referred to a tertiary center for prenatal care in order to significantly reduce adverse birth outcomes such as PTBs. Social marketing approaches emphasizing the need to seek early and focused antenatal care, aimed at men and women planning to become parents, will be a powerful strategy to reduce cases of complicated non-booked mothers and PTBs. Another upstream approach is to establish early in pregnancy a referral system between peripheral service providers, private practitioners, TBAs, and nearby specialist hospitals to ensure that every woman gets quality prenatal as well as antenatal care. This may not be possible without incentives for participants and therefore requires the establishment of a cluster of health care providers and a rewards system. Nonetheless, our data as well as evidence from other studies clearly show that un-booked mothers are at the greatest risk for PTBs and we must find ways of addressing this using all possible strategies.

**Supplementary information**: data for this study was obtained from the labor ward records of the Lagos University Teaching Hospital (LUTH), Surulere, Lagos, Nigeria following data use agreement between the University of Iowa and the University of Lagos. We also obtained non-human subject Institutional Review (IRB) approval from the University of Iowa (IRB approval number 201406748). Information was retrieved from delivery room registry for deliveries that took place between January 2011 and December 2013. This information was entered into an Excel spreadsheet. The information was then imported, de-identified, into a secured online Redcap database [42].

### What is known about this topic

- It has been well established that preterm birth is a pregnancy complication that increases risk for morbidity and mortality with a huge financial burden globally;
- It is a known fact that Nigeria has the highest number of newborn deaths in Africa, and the second highest in the world after India about 270,000 children in Nigeria per year die within the first month of life as a direct consequence of ptb and low birth weight, perinatal asphyxia and infections;
- It is well known that the degree of prematurity inversely relates to the likelihood of increased mortality and morbidity.

### What this study adds

- This study adds to the body of evidence that the rate of PTB is significantly high in Nigeria and we provide striking evidence that exists at both ends of the spectrum as it relates to provision and utilization of perinatal care;
- This study recommends that every pregnancy that is regarded as high risk by the health care system in Nigeria be referred to a tertiary center for prenatal care in order to significantly reduce adverse birth outcomes such as PTBs;
- Social marketing approaches emphasizing the need to seek early and focused antenatal care, aimed at men and women planning to become parents, will be a powerful strategy to reduce cases of complicated non-booked mothers and PTBs.
